# Stakeholder perceptions of non-regulatory bovine health issues in Ireland: past and future perspectives

**DOI:** 10.1186/s13620-020-00178-8

**Published:** 2020-11-26

**Authors:** Natascha V. Meunier, Kenneth McKenzie, David A. Graham, Simon J. More

**Affiliations:** 1 Animal Health Ireland, Carrick-on-Shannon, N41 WN27 Ireland; 2 Department of Management, School of Business & Humanities, TU Dublin, Tallaght, Ireland; 3 UCD Centre for Veterinary Epidemiology and Risk Analysis, School of Veterinary Medicine, University College Dublin, Dublin, D04 W6F6 Ireland

**Keywords:** Non-regulatory bovine health issues, Cattle, Prioritisation, Ireland

## Abstract

**Background:**

In recent years, there have been multiple (political, environmental, cultural) drivers of change in Irish agriculture, including the establishment of Animal Health Ireland (AHI) in 2009, to provide leadership of non-regulatory livestock health issues (diseases and conditions of livestock that are endemic in Ireland but which are not currently subject to international legislation). In this study, we describe the opinion of stakeholders (farmers, veterinary practitioners and agricultural industry professional service providers), elicited by means of a survey, on their perceptions of changes in selected non-regulatory bovine health issues over the last 10 years and priority issues relevant to non-regulatory bovine health to be tackled over the next 10 years.

**Results:**

A total of 673 individuals participated in the online questionnaire. For the majority of the non-regulatory bovine health issues, most participants felt there had been improvements over the last 10 years. However, professional service providers were generally more conservative in their response to improvements on-farm compared to farmers. Several issues, particularly BVD and udder health/milk quality, were viewed more positively by all relevant respondents. There was reasonable agreement between responses from different respondent types and sectors regarding the top three priorities relevant to non-regulatory bovine animal health for the next 10 years in Ireland, which included antimicrobial resistance (highlighting measures to reduce both on-farm usage and resistance), anthelmintic resistance, greenhouse emissions and calf welfare.

**Conclusions:**

The results are encouraging, demonstrating a perception of improvement in a number of non-regulatory bovine health issues in Ireland over the last ten years. With respect to the next 10 years, stakeholders prioritised antimicrobial and anthelmintic resistance, greenhouse gas emissions and calf welfare, which aligns closely with broader societal concerns. This information is useful to AHI, particularly with respect to future priorities. However, these concerns are broad in scope and will require further considerations, including collaborations, between AHI and partnering organisations. Given that there were differences between farmers and professional service providers in responses, it is useful to consider how the aims and the benefits of future AHI programmes are framed and communicated to all stakeholders.

**Supplementary Information:**

The online version contains supplementary material available at 10.1186/s13620-020-00178-8.

## Background

Animal health forms a key part of the daily work of the Irish government, through the national Department of Agriculture, Food and the Marine (DAFM), under the direction of the Chief Veterinary Officer. DAFM’s work focuses on policy development, supporting farm productivity, ensuring food safety and regulating to ensure Ireland’s compliance with EU and Irish legislation relating to critical areas such as bovine tuberculosis [35], exports, animal welfare and transboundary diseases. Since 2009, leadership of non-regulatory animal health issues (diseases and conditions of livestock that are endemic in Ireland but not currently subject to international legislation [7];) has been the responsibility of Animal Health Ireland (AHI), an industry-led, not-for-profit organisation that operates as a partnership between livestock producers, processors, animal health advisors and government [33]. The organisation aims to increase the profitability and sustainability of farming enterprises and to enhance the competitiveness of Irish products in the international marketplace through improvements in animal health and welfare [7].

There is an increasing shift by state organisations, quasi-autonomous state organisations, and hybrid organisations (blending state, profit, and not-for-profit representatives within any one sector) towards stakeholders, to help inform organisational decision-making and action-taking [21]. AHI is an example of a hybrid organisation, reflecting the statutory, for-profit, and not-for-profit bases of farming in Ireland. From its inception in 2009, AHI recognised the need to be informed by the full breadth of perspectives on bovine health in Ireland, and to balance these perspectives to help inform its programme of work. This ‘bias towards consultation’ was deemed essential as it was easy to imagine scenarios whereby one group’s view effectively contradicted another’s and would lead to a logjam. One partial solution to this likely problem that AHI developed was to elicit stakeholder views on what were perceived to be the main issues in bovine health and how these issues should be prioritised, and to write up the results of this perspective-gathering exercise in academic journals, as a demonstration of AHI’s commitment to transparency. It was hypothesised that this proof of transparency would encourage all stakeholders to acknowledge that AHI was evenhanded in how it approached its bovine health programme development, thus facilitating open exchanges of views, constructive debate, and the fullest possible level of agreement on AHI’s programme development.

Shortly after AHI’s establishment in 2009, studies were conducted seeking expert (Delphi policy study) and farmer (priority identification surveys) opinions on bovine health issues of importance to guide subsequent national action. Key priority issues identified included both disease-specific (BVD, IBR, paratuberculosis) and syndromic (udder health/milk quality, lameness, diseases of young calves, fertility) conditions. Beef farmers additionally prioritised parasitic conditions and weanling pneumonia [36]. This consultative exercise informed the five national programmes that were subsequently developed by AHI, namely the bovine viral diarrhoea (BVD) national eradication programme, the CellCheck national mastitis control programme, the Irish Johne’s control programme, Beef HealthCheck health monitoring at slaughter, and the infectious bovine rhinotracheitis (IBR) programme. In most cases, each programme was supported by a technical working group (to provide scientific perspectives) and an implementation group (to guide policy development and action), each underpinned by agreement and consensus. A further three work areas were developed by AHI with a particular focus on farmer and veterinary practitioner education, relating to calf care and welfare (CalfCare), biosecurity, and parasite control. Over the last 10 years, substantial progress has been made across the AHI portfolio of bovine health programmes, as summarised in Table 1.

**Table 1:** Animal Health Ireland’s portfolio of bovine health programmes, including national key indicators and programme achievements

Programme	Health focus	Date began	National key indicators	Programme achievements	Reference
BVD national eradication programme	Bovine viral diarrhoea	Voluntary 2012, compulsory 2013	- Decrease of persistently infected calves from 0.66% in 2013 to 0.04% in 2019 - Decrease in proportion of positive breeding herds from 11.30% in 2013 to 0.77% (390 of 83,000) in 2019	- Estimated farmer annual net saving of € 85 million in 2019	[2, 40]
Irish Johne’s Control Programme	Johne’s disease *(Mycobacterium avium* subspecies *paratuberculosis)*	Pilot (dairy) 2013, Voluntary (dairy) 2017	- Estimated prevalence in 2005 at herd level was 21.45% (95%CI 18.4–24.9) and at the animal level was 2.86% (95%CI 2.76–2.97)	- 9% of dairy herds participating in 2019, representing 11% of dairy cattle	[2]
CellCheck	Mastitis	2012	- Reduction in national average somatic cell count (SCC) from 234,000 in 2013 to 176,000 in 2019 - Reduction in estimated on-farm antibiotic usage, measured in terms of defined course dose per cow per year, both for dry cow therapy (34% reduction) and in-lactation therapy (15% reduction) during 2013–18, although dry cow therapy has increased subsequently	- Associated economic benefit to farmers and processors estimated at €39 million and €16 million, respectively, in 2017	[22, 2, 23, 35] McAloon, McCoy, and More, n.d.) *L. Shalloo, unpublished data*
Beef HealthCheck	Liver fluke and pneumonia at slaughter	2016	- Programme prevalence in 2019 of liver fluke damage or live fluke at slaughter was 11.5% at the animal level and 57.3% at the herd level.	- Farmer and veterinary practitioner access to individualised slaughter reports through a programme database presenting herd- and practice-level dashboards - Data captured on 717,000 cattle and 27,000 herds in 2019 - 17 participating abattoirs - Annual farmer educational events - Economic impact of fluke on steer production investigated - Incorporation of genetic resistance to liver fluke into dairy and beef breeding indices	[2, 10, 43, 44, 26, 42]
IBR	Infectious bovine rhinotracheitis	Pilot 2018	- Herd level seroprevalence of 74.9% (95%CI 69.9–79.8%) in 2009. - Beef herd prevalence was estimated at 90% in 2014/15. - Increase in IBR vaccine sales of 12.3% between 2017 and 2019	- Pilot control programme - Preparations for a national control programme - Commencement of national bulk tank surveillance testing - Information leaflets	[13, 9, 2, 5]
CalfCare	Diseases and welfare of calves	2010		- Information leaflets on best practice - Annual farmer educational events	[4]
Parasite Control	Endo- and ectoparasites	2010		- Information leaflets on best practice	[6]
Biosecurity	Biocontainment and bioexclusion	2010		- Information leaflets on best practice	[3]

In the future, AHI will face a changing external environment (including economic, environmental and regulatory drivers, ongoing organisational issues relating to strategic planning, resource constraints (budgetary, personnel) and a broadening scope (for example, the inclusion of additional animal species). Irish agriculture is currently facing multiple challenges relating to farm profitability, ongoing reform of the Common Agricultural Policy (CAP), structural changes following removal of milk quotas in 2015, climate change and the national commitment to reduce greenhouse gas emissions [16, 28], and negative impacts as a consequence of Brexit [12]. In addition to economic and environmental challenges, a series of policies relating to animal health and welfare will also impact Irish farming, including EU legislation (particularly the Animal Health Law [19], the Veterinary Medicinal Products Regulations [20]) and national legislation and initiatives (Ireland’s National Action Plan for Antimicrobial Resistance 2017–2020 (iNAP) [17], National Farmed Animal Health Strategy [14].

It was against this backdrop of political, environmental and cultural change that AHI decided to approach stakeholders to seek their views on how they perceived bovine health issues had changed (if at all) over the last 10 years, and to get their sense of issues on the horizon that are likely to have significant implications for farming as an activity in Ireland. Two necessary caveats must be attached here. Firstly, by farming activity we mean farming activity as perceived by those in dairy and beef farming. Of course, farmers and farm stakeholders come across people whose livelihoods come from other parts of the farming sector, so there is inevitably some mixing of views from direct experience (the farmers’ own farms; the stakeholder’s own working lives), and those obtained through indirect experience (their interactions with people in farming, but outside dairy and beef farming; their interpretation of news on farming as a whole). The second caveat is that there is likely to be a heterogeneity of interpretations when we ask farmers and stakeholders (and indeed anyone) for what they think is likely to be important for their sector over the coming years. The elicitation exercise here should therefore be seen as an attempt to canvass opinion and to use such opinion to help inform programme development for AHI. It should not be seen as an exercise in futurology, which has its own methods and toolkits.

As in 2009, AHI again chose a prioritisation exercise as the way to canvass opinions. Prioritisation exercises have been used in a number of different settings and using different methods. Examples include prioritisation of non-regulatory bovine health issues in Ireland [36], orphan drug regulation in Europe [37] and wildlife pathogens to be targeted for surveillance [11]. Further, there are several examples where expert and farmer opinions were compared, relating to non-regulatory animal health issues [36, 46], land use suitability [39] and agricultural greenhouse gas emissions [27].

Here, we describe the opinion of Irish stakeholders (farmers and professional service providers), elicited by means of a survey, on their perceptions of changes in selected non-regulatory bovine health issues over the last 10 years and priority issues relevant to non-regulatory bovine health that need to be tackled over the next 10 years.

## Materials and methods

### Survey content

It is important to stress that AHI wanted to maintain its ‘bias towards consultation’ throughout this prioritisation exercise. Consequently, there was an emphasis on free text ‘open’ responses, as well as a ranking system for focus areas that fell within AHI’s remit since its inception.

The survey was composed of two main sections: changes on-farm in the last ten years in selected non-regulatory bovine health issues (specifically, those aligned with current AHI programme areas), and potential priorities for the next 10 years. The survey was distributed to farmers and professional service providers. For both sections, farmers were asked to relate to changes and priorities *on their farm*, whereas professional service providers were asked to relate to changes and priorities *on the typical Irish farm* for each sector. The farming industry was further divided into three sectors: dairy, beef suckler and beef fattening/finisher. In Ireland, suckler farmers would typically be involved in breeding whereas beef fattening/finisher systems are typically non-breeding herds. Variations exist in individual herds in both systems for the age at which animals are bought or sold and either system may send animals to slaughter. Farmers were asked to comment only on the sector they are most associated with, whereas professional service providers were asked to separately comment on all three cattle sectors.

In the first section of the survey, the following eight non-regulatory bovine health issues were considered: bovine viral diarrhoea (BVD), infectious bovine rhinotracheitis (IBR), Johne’s disease (paratuberculosis), udder health/milk quality, monitoring/feedback of liver and lung lesions at slaughter, diseases of young calves, farm biosecurity, and parasite control. Separately for each of these non-regulatory bovine health issues, participants were asked to indicate either their opinion of the change over the last 10 years in the health status of cattle on their own farm (farmers), or a typical Irish farm within each sector (professional service providers). Answers were captured using a four-point Likert-type scale, with options to indicate the health status as being ‘much worse’, ‘somewhat worse’, ‘somewhat better’ or ‘much better’. An additional option of ‘Don’t know/not relevant’ was given to encourage respondents to weigh up the merits and demerits of each health issue. Options relating to diseases of young calves, Johne’s disease, and udder health/milk quality were not presented to responders relating to the beef fattening/finisher sector. Udder health/milk quality was also not relevant in this context to suckler farmers and therefore was not presented to them.

In the second section of the survey, participants were asked to select, from a predefined list, up to three priority issues relevant to non-regulatory bovine animal health that in their view needs to be tackled on their (for farmers) or a typical Irish (for professional service providers) farm over the next 10 years. This list was formulated on the outcomes of the previous Delphi policy study [36], expert opinion and stakeholder discussions, as well as emerging government and EU policy changes. The predefined list included:

- antibiotic resistance (measures to reduce on-farm usage and antibiotic resistance), *hereafter referred to as antimicrobial resistance in this paper,*
- anthelmintic resistance (measures to reduce resistance to fluke and worm treatments),
- greenhouse emissions (measures to reduce emissions through health-driven efficiencies),
- calf welfare,
- lameness,
- infertility,
- *Mycoplasma bovis*,
- clostridial diseases.

By a process of logical elimination, calf welfare and infertility were not presented as specific items to the beef fattening/finisher sector.

Open text responses were elicited for any further priorities that the participant felt should be among the top three priorities. A copy of the survey is available in the additional files.

### Study population

The intention of the survey was to elicit opinion widely within the livestock industry in Ireland, both from farmers and professional service providers. Farmers within the HerdPlus programme of the Irish Cattle Breeding Federation (ICBF) were targeted for the survey. From a convenience sample of ICBF farmers registered to HerdPlus and with email addresses on file, 3117 farmers were randomly selected and contacted to participate in the survey. The sample of farmers was confirmed to be representative in terms of both herd size and associated sector of their herds in the database. This sample was 18% of the herds in the HerdPlus database with an email address and represented 3.1% of the reported 100,767 cattle herds in Ireland, of which 28,994 are estimated to be non-breeding herds [15]. Several groups of professional service providers were targeted. Private veterinary practitioners that were listed within the AHI database were contacted directly and further distribution was made through Veterinary Ireland and the Progressive Veterinary Network. State veterinarians were contacted through DAFM as well as the Veterinary Officers’ Association. Agricultural service providers were contacted through Teagasc, the Agricultural Consultants’ Association, Agricultural Science Association and Farm Relief Services. In all stakeholder cases, there was an element of reliance on a snowball sampling method, in that it was hoped that survey participants would share the link with others in their circle. The survey was also distributed at the UCD School of Veterinary Medicine to staff of the Veterinary Pathobiology and Herd Health and Animal Husbandry divisions, as well as to final year undergraduate veterinary students and to postgraduate students with a cattle-focused research interest. Members of the AHI implementation and technical working groups were contacted directly.

### Survey administration

The survey was made available through an online platform (www.typeform.com). Farmers and professional service providers were contacted in June 2019 via email with a link to complete the online questionnaire. The questionnaire was further highlighted on the AHI website and social media with a link that remained active throughout the study period. A follow-up email was sent after 2 weeks. The survey was closed to new responses after six weeks. All responses were anonymised and no personal information was captured during the survey. Multiple responses from the same computer were prevented by the platform.

### Analysis

Descriptive analysis of the results was performed with R statistical software [38]. In presenting the opinions of selected non-regulatory bovine health issues, the ‘much worse’ and ‘somewhat worse’ response categories were combined into a single ‘worse’ category. The results are presented by the three broader sector categories: dairy, suckler and beef finisher/fattener, and by type of respondent (either farmer or professional service provider).

For the future priorities, a weighted ranking system was used to identify the ranking order. Twelve points were distributed among the preferences chosen per respondent, such that if only one priority was chosen, 12 points were awarded to that preference. If two options were chosen, each preference was awarded 6 points, and likewise 4 points were awarded to each of three preferences chosen. Each additional preference highlighted in the open text was awarded 1 point. Such weighting is an attempt to take account of what participants truly considered to be priorities.

## Results

### Respondents

A total of 673 people filled in the online questionnaire, including 410 (60.9%) farmers, of whom 234 categorised themselves as beef suckler, 145 dairy, and 31 beef fattener/finisher farmers. The 231 professional service provider respondents included private veterinary practitioners (*n* = 88, 13.1%), state veterinarians (*n* = 73, 10.9%), 50 (7.4%) agricultural advisory, consultancy or industry personnel, 19 (2.8%) UCD School of Veterinary medicine staff and students, and 1 individual identifying as farm relief. A further 32 (4.8%) respondents identified as ‘other’. Among the professional service providers, 77 also identified as a member of one of AHI’s implementation groups or technical working groups.

The overall response rates for farmers was 13.2%, with a slightly higher response from dairy farmers (14.8%) compared to beef farmers (13.5%). A response rate of 10.3% from private veterinary practitioners was received. A 13.6% response rate was seen from emails distributed within the UCD School of Veterinary Medicine. Response rates could not be calculated for individuals in other sectors as key individuals within organisations were asked to distribute the survey as widely as possible and therefore denominators were not recorded.

### Past perspectives (the last 10 years)

**Fig. 1:**
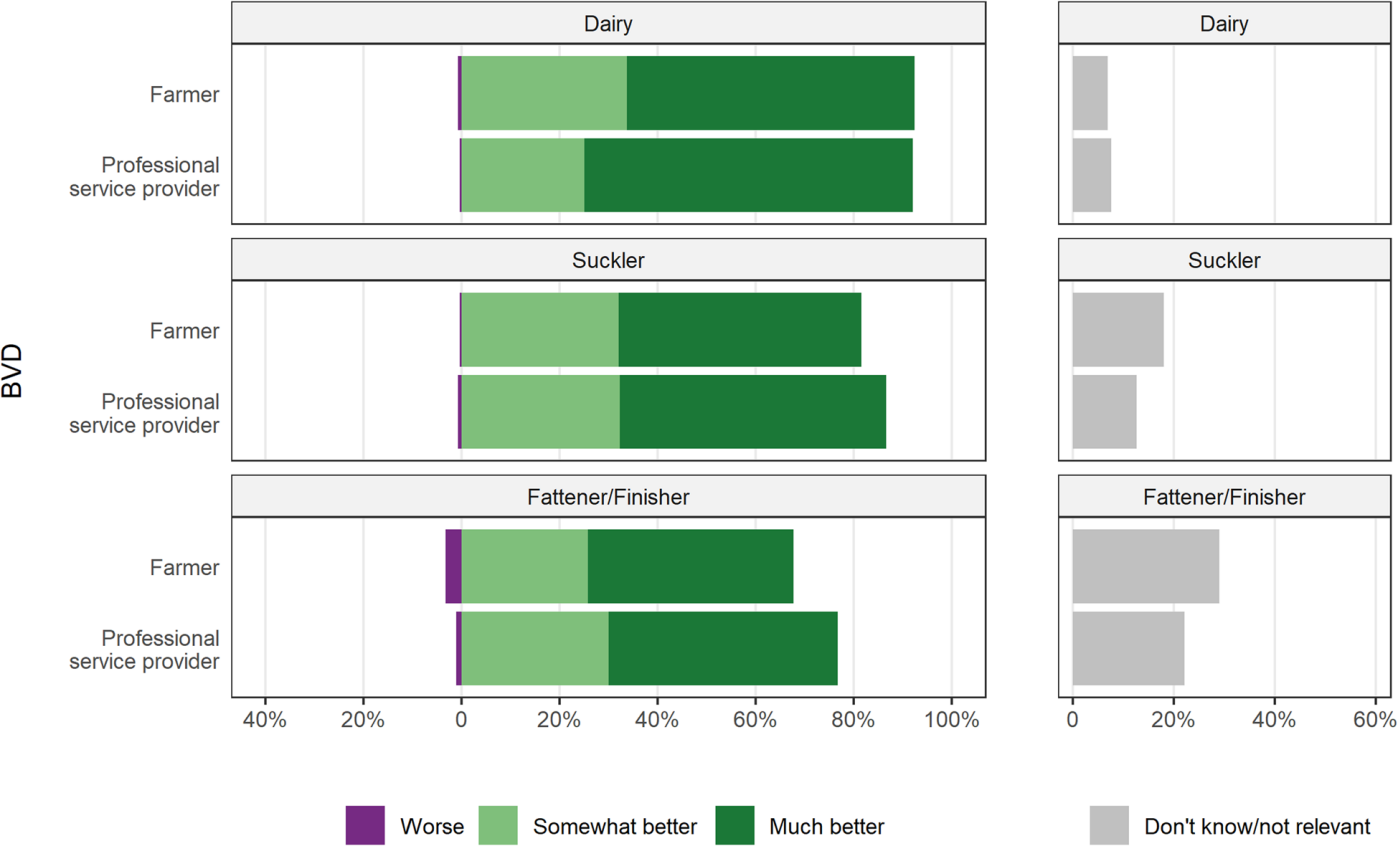
Opinions of Irish farmers and professional service providers of the change over the last 10 years in the BVD health status of cattle, either on their farm (for farmers) or the typical Irish farm (for professional service providers), by sector and type of respondent. For this question, farmers were asked to comment only on the sector they are most associated with, whereas professional service providers were asked to separately comment on all three sectors, dairy, beef suckler and beef fattener/finisher

**Fig. 2:**
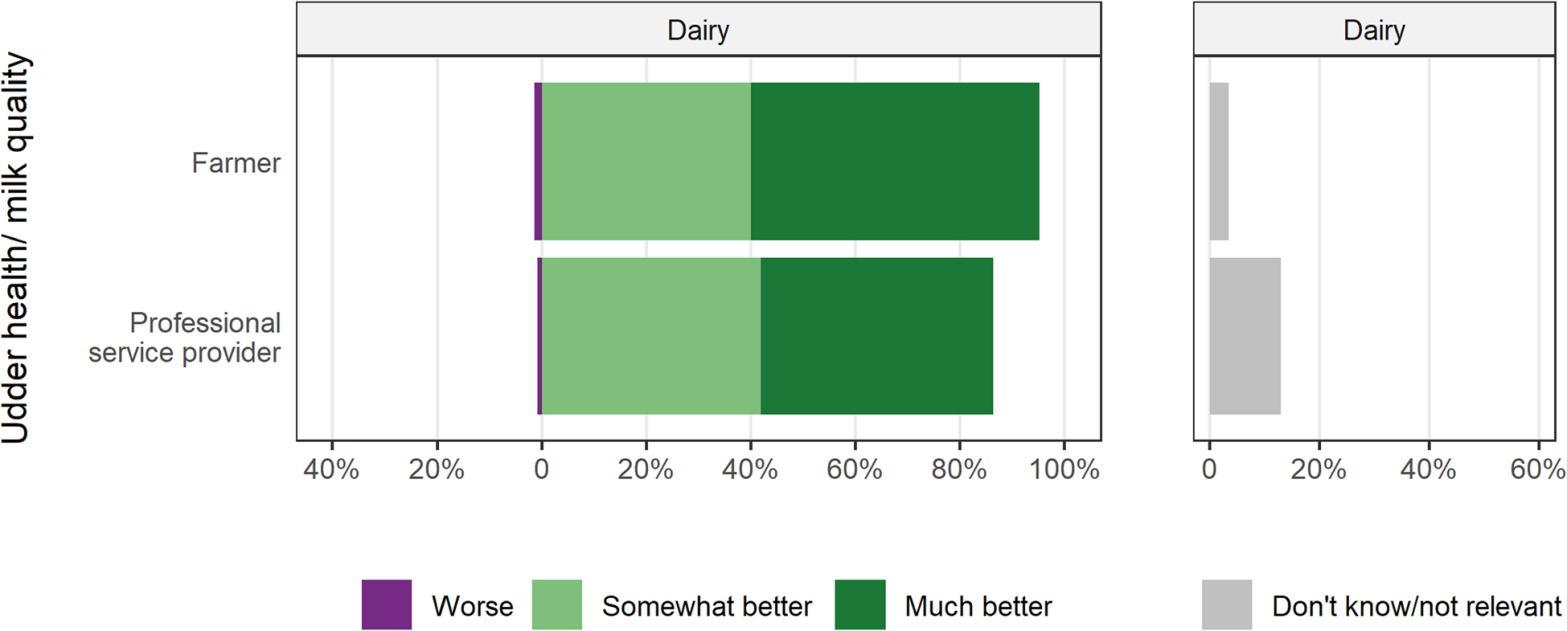
Opinions of Irish farmers and professional service providers of the change over the last 10 years in the udder health/milk quality status of cattle, either on their farm (for farmers) or the typical Irish farm (for professional service providers), by type of respondent. For this question, dairy farmers and all professional service providers are represented

For the majority of the non-regulatory bovine health issues, most participants felt there had been improvements over the last 10 years. However, professional service providers were generally more conservative in their response to improvements on-farm compared to farmers. The exceptions to this were for BVD and udder health/milk quality where 41–67% of farmers and professional service providers agree to a ‘much better’ animal health status and at most, 3% felt the situation had become worse (Figs. 1 and 2). Considering both ‘somewhat better’ and ‘much better’ responses for BVD, 68 to 92% of farmers and professional service providers in all sectors felt the animal health status now belonged in these categories. Between 22 and 29% of beef fattener/finishers respondents either did not know or felt that the BVD status of animals did not apply to their situations. Regarding udder health/milk quality, 95% of farmers and 86% of professional service providers reported an improved situation.

**Fig. 3:**
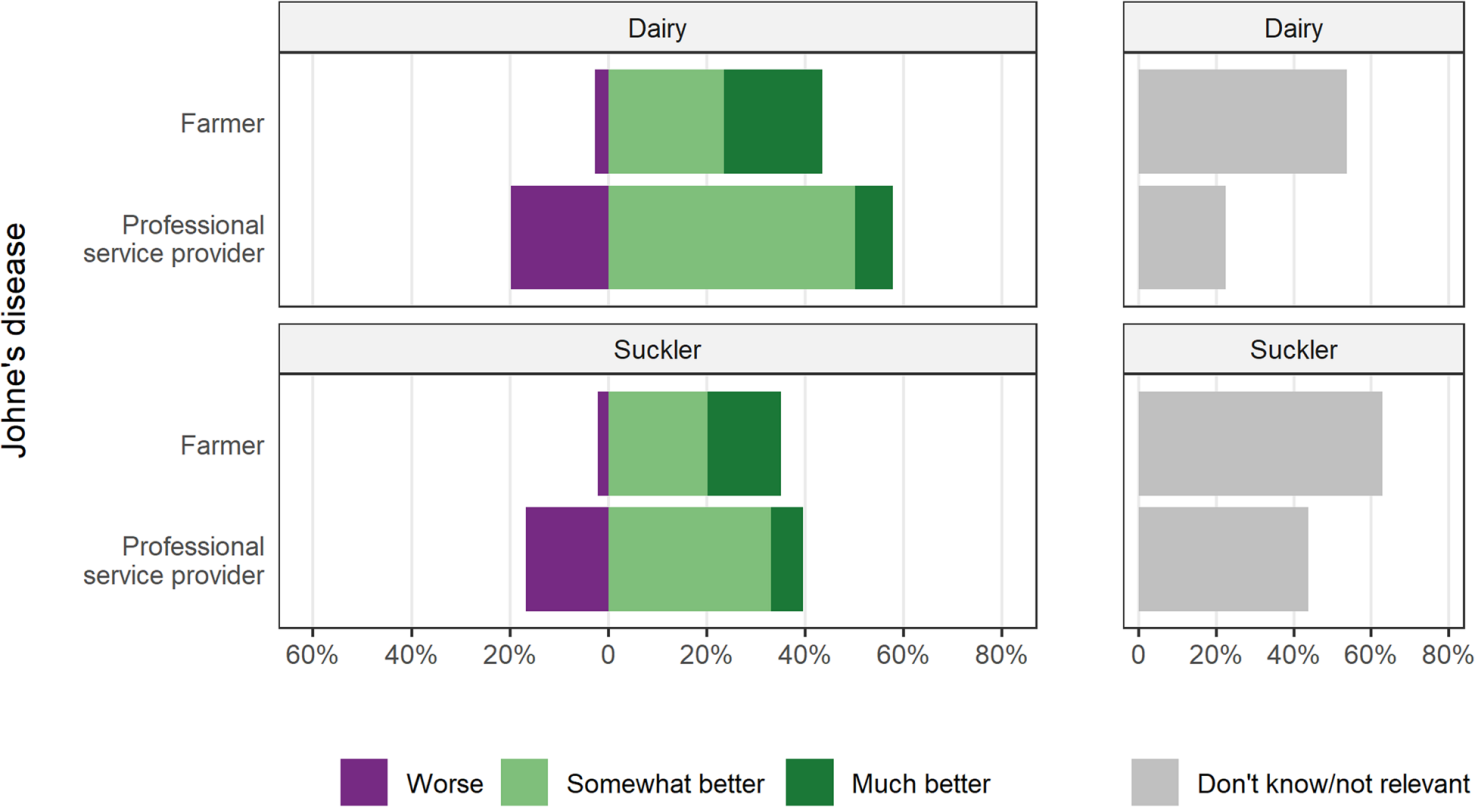
Opinions of Irish farmers and professional service providers of the change over the last 10 years in the Johne’s disease health status of cattle, either on their farm (for farmers) or the typical Irish farm (for professional service providers), by sector and type of respondent. For this question, farmers were asked to comment only on the sector they are most associated with, whereas professional service providers were asked to separately comment on both the dairy and beef suckler sectors

Opinions about Johne’s disease were asked only with respect to the dairy and suckler sectors, with at most 50% of professional service providers suggesting that health status was ‘somewhat better’ (Fig. 3). Only 20% of dairy farmers and 15% of suckler farmers, among those who responded, felt the Johne’s disease health status on their farm was ‘much better’, while for professional service providers this was below 8%. Some professional service providers felt that the situation had become ‘worse’ in the dairy (20%) and suckler (17%) sectors. Many farmers in the dairy (54%) and suckler (63%) sectors either did not know or felt that Johne’s disease was not relevant.

**Fig. 4:**
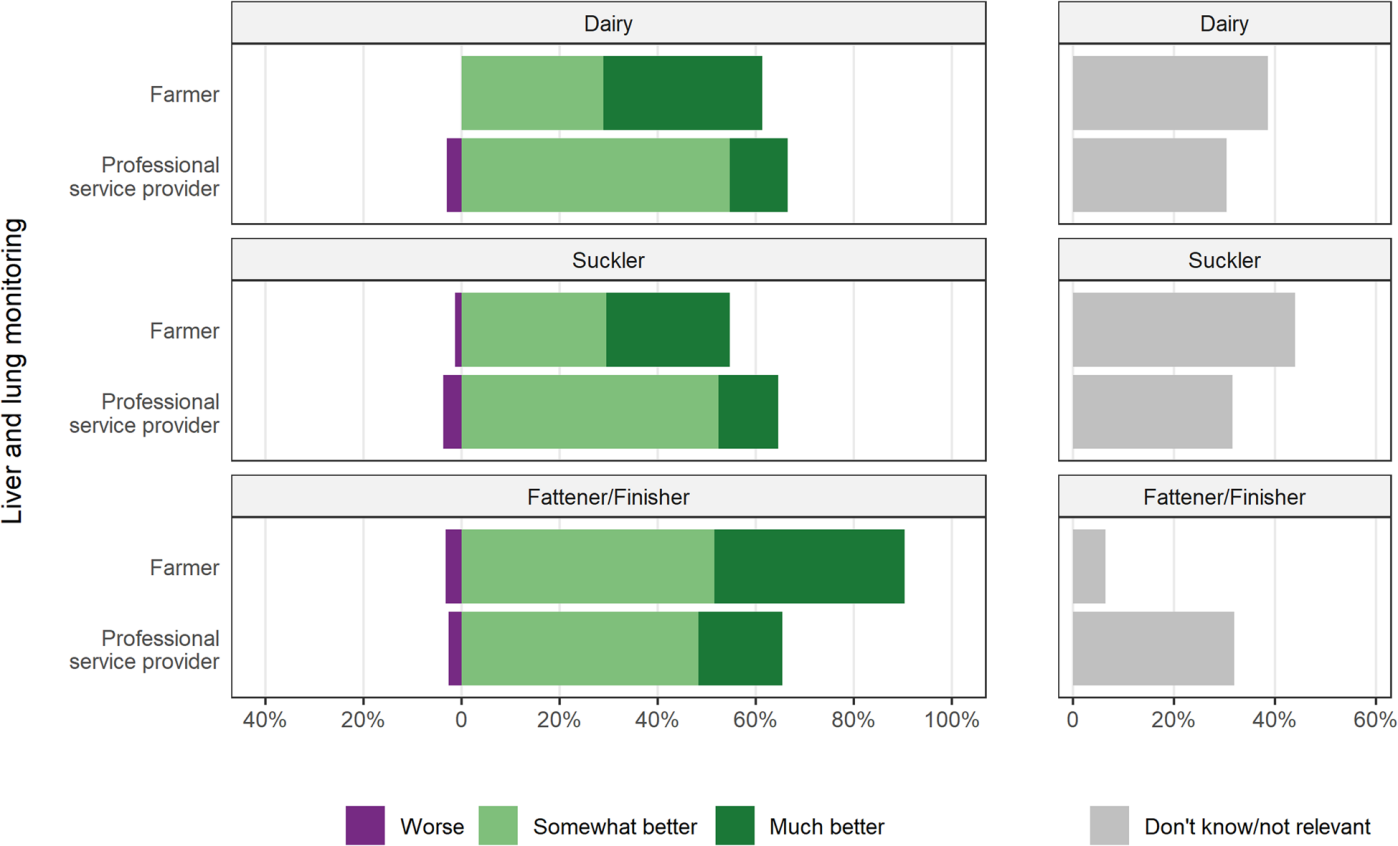
Opinions of Irish farmers and professional service providers of the change over the last 10 years in the status of cattle with respect to liver and lung lesions at slaughter, either on their farm (for farmers) or the typical Irish farm (for professional service providers), by sector and type of respondent. For this question, farmers were asked to comment only on the sector they are most associated with, whereas professional service providers were asked to separately comment on all three sectors, dairy, beef suckler and beef fattener/finisher

The status of cattle with respect to liver and lung lesions at slaughter was considered ‘much better’ by 39% of the beef fattener/finisher farmers, 32% of dairy and 25% of suckler farmers, with 90% of beef fattener/finisher farmers seeing a ‘somewhat better’ or ‘much better’ status (Fig. 4). The majority of professional service providers (48 to 55%) felt the situation was ‘somewhat better’.

With respect to diseases of calves, parasite control and biosecurity, 44 to 58% of farmers felt the situation of their farm was ‘much better’. Similarly, for 31 to 42% of farmers, the IBR status was felt to be ‘much better’. Professional service provider responses were more measured, with the majority falling under ‘somewhat better’ for the typical Irish farm. A summary of the responses on opinions for all programmes is available in Additional File 2.

### Future perspectives (the next 10 years)

There was reasonable agreement between responses from different respondent types and sectors about the top three priorities relevant to non-regulatory bovine animal health for the next 10 years in Ireland, considering either their own farm (farmers) or a typical Irish farm (professional service providers). Antimicrobial resistance (highlighting measures to reduce on-farm usage and antibiotic resistance) was ranked first by farmers and professional service providers with respect to the dairy and beef fattener/ finisher sectors, and also received a first or second ranking with respect to the beef suckler sector (Table 2). Anthelmintic resistance ranked within the top three and greenhouse emissions were ranked either third or fourth, with the exact ranking differing by sector respondent types. Calf welfare was ranked in the top four by all respondents except dairy farmers (fifth). Consistent with this, calf welfare was ranked as a higher priority by professional service providers than by farmers when considering responses for the dairy sector while the converse was the case in the suckler sector.

**Table 2:** Future AHI priorities ranked by sector and respondent group (1 highest ranking, 8 lowest ranking). The top three ranked priorities are in bold. Calf welfare and infertility were not asked of the fattener/finisher sector

Priority	Dairy		Suckler		Fattener/ Finisher	
	Farmer	Professional service providers	Farmer	Professional service providers	Farmer	Professional service providers
Antimicrobial resistance	**1**	**1**	**2**	**1**	**1**	**1**
Anthelmintic resistance	**2**	**3**	**1**	**2**	**2**	**2**
Greenhouse emissions	**3**	4	4	**3**	**3**	**3**
Calf welfare	5	**2**	**3**	4	–	–
Infertility	4	6	5	5	–	–
Lameness	6	5	7	8	5	4
Clostridial diseases	8	8	6	6	4	6
*Mycoplasma bovis*	7	7	8	7	6	5

Antimicrobial resistance, anthelmintic resistance and greenhouse gas emissions were consistently ranked as the top three priorities for all stakeholders in the beef fattener/finisher sectors.

## Discussion

### Perceptions on health changes

This study presented the opinion of farmers and professional service providers (including veterinary practitioners and agricultural industry professionals) on non-regulatory bovine health concerns in Ireland. The study looked at health concerns that directly reflected the work areas of AHI (Table 1). However there are multiple actors influencing the industry, which itself is constantly evolving. While some changes can be attributed to the work of AHI, the aim of this study was not an evaluation of the AHI programmes, which is not appropriate for this type of survey, but rather to gather the perception of changes that have occurred.

The largest driver for change in the dairy industry, the abolition of milk quotas, provided an incentive for an increase in efficient production, allowing dairy farming to become more profitable in the Irish context. Ireland saw an increase in the average dairy herd size over last ten years from 64 animals in 2010 to 80 animals in 2019, with a corresponding increase in milk production per hectare [18]. These changes were supported by Knowledge Transfer programmes and advisory services developed and delivered by a range of players, including DAFM, the Agriculture and Food Development Authority (Teagasc) and AHI. These provided direct communication of best practice to farmers, with ongoing discussion groups nationally in both beef and dairy, at regular meetings discussing improvements in technical, financial and scientific knowledge. The last decade also saw major development of genetic evaluations and improvements in the quality of the national herd by the Irish Cattle Breeding Federation (ICBF). While these efforts were initially focused on the dairy herd, progress has also been made in beef breeds. Breeding and economic evaluations have been developed for the selection of genetically superior animals with improvements of quality and performance such as fertility, calving ease and more recently, the health conditions bovine tuberculosis and liver fluke. The AHI programmes have been operating alongside, often in collaboration with, these other developments, exerting variable direct influence on these bovine health outcomes.

There was broad agreement across sectors and respondents with regard to BVD, recognising that there had been substantial improvement. This mirrors the technical progress made to date at the farm level from 2013 to 2019 with herd prevalence decreasing from 11.3 to 0.77% and animal prevalence decreasing from 0.66 to 0.04% during this period (Table 1). The slightly lower rankings on BVD from all respondents regarding the fattening/finishing sector may reflect the fact that the eradication programme is addressed primarily to breeding herds. While fattener/finishing herds are beneficiaries of this programme through the removal of persistently infected animals before they reach this production stage, this appears not to be fully acknowledged.

Udder health and milk quality were also viewed positively by all respondents within the dairy sector. The AHI CellCheck programme has contributed to this issue, providing a national focus agreed by all stakeholders towards a substantial improvement in national milk quality, as measured through bulk milk tank somatic cell count (SCC), and reduced intramammary antimicrobial usage, both at drying off and during lactation [34]; C.I. McAloon, personal communication). The increase in efficient milk production has been an important incentive for a decrease in SCC and mastitis, as this would complement quality and efficiency of production. Additionally, messaging around colostrum intake has been widely promoted by AHI and the knowledge exchange programmes to improve calf welfare and health. The decreased prevalence of BVD would have also influenced calf health positively.

In contrast, Johne’s disease was reported as showing less improvement, with professional service providers being less positive in their responses than farmers. These views are reasonable given that the lack of national agreement, until very recently, about appropriate strategies for national Johne’s disease control. The Irish Johne’s Control Programme, a voluntary programme coordinated by AHI, was launched in 2017 following substantial planning and research, and has subsequently been implemented in a relatively limited number of Irish dairy herds. There is some evidence of increasing herd prevalence of Johne’s disease in Ireland over the last several decades [24, 30], although accurate measurement of herd prevalence has proved challenging [31]. A number of reasons have been identified facilitating dissemination of infection within Irish herds, including the introduction of many animals with the national move to the single EU market in 1992 (affecting both dairy and pedigree beef breeds for improved performance traits) [8] and substantial levels of within-country movement [32]. Other challenges are not country-specific, including the use of imperfect diagnostic tests, a prolonged disease course and sub-optimal farm biosecurity [24]. Over the last 10 years, there have been concerted efforts towards improved farmer awareness of these issues, in part through AHI’s biosecurity activities and Irish Johne’s Control Programmes, and from dairy processors and within Teagasc discussion groups.

The monitoring and feedback of liver and lung lesions at slaughter, directly related to AHI’s Beef HealthCheck programme (Table 1), was also viewed differently by farmers and professional service providers, being viewed very positively by farmers, particularly those in the fattener/finisher sector. Reports from Beef HealthCheck are delivered directly to farmers on an individual basis, for on-farm decision making, with more generalised results aimed at a wider audience, which would include professional service providers, where they may lose apparent utility. The highest ranking from farmers in the fattener/finisher sector may reflect the fact that the programme is particularly relevant to herds that are slaughtering larger numbers of cattle.

### Perceptions on prioritisation

This study provides stakeholder insights into priority issues relevant to non-regulatory bovine health that need to be tackled over the next 10 years. These issues, which are in general agreement with broader societal concerns, include resistance by pathogenic organisms to medications, including both antimicrobial resistance against bacterial infections and anthelmintic resistance for treatments against parasitic infections; greenhouse gas emissions; and animal welfare incorporating calf and cow welfare, as well as lameness specifically.

Improvements in animal health, for example BVD eradication, improved milk quality and reduced parasite load, are associated with production efficiencies, and therefore the potential for equivalent output from fewer animals and reduced greenhouse gas emissions. This was highlighted in a study by Williams et al. [45], who reported the potential for 2–5% reductions in greenhouse gas emissions with improved animal health. Improving animal health and economic breeding indices are mitigation measures with regard to agricultural methane by improving animal production efficiency [1, 28]. Indeed, the recently revised Marginal Abatement Cost Curve for Irish agriculture [28] shows that animal health measures have a contribution to make to greenhouse gas abatement, contributing an annual average mitigation over the next ten years of 147ktCO_2_e. This is broadly similar to that achieved by improved nitrogen use efficiency or low emission slurry spreading and represents some 7.3% of the total average annual savings. Furthermore, these savings are shown to be cost-beneficial in terms of achieving this abatement.

An understanding of these priority areas is relevant to the ongoing work of AHI, which provides national leadership in non-regulatory bovine health issues. However, these issues are complex, requiring a more sophisticated and collaborative response than was required when considering animal health alone and indeed an issue attributable to a single pathogen. That said, these identified future priorities are already being addressed, at least in part, through ongoing national work, including by AHI. For example, BVD eradication contributes to reduced on-farm antimicrobial usage by mitigating the cohort-level immunosuppressive impact of BVD, particularly in younger animals [25, 29]. Further, herds with reduced SCC have fewer clinical and subclinical cases of mastitis and therefore less need for in-lactation intramammary antimicrobial usage and also have a greater potential to transition from blanket to selective dry cow therapy without excessive risk [34].

AHI will need to continue to work collaboratively with other organisations (noting, for example, a very broad multi-agency response to antimicrobial resistance), taking the lead in specific areas where appropriate. Recent strategies, for example the antimicrobial work in support of iNAP [17], and the convening by DAFM of an Antiparasitic Resistance Stakeholder Group (of which AHI is a member), represents an ongoing shift towards integrated, multiagency action on complex issues. A follow-on to this survey involving a consultative process will be particularly important to identify how AHI can best contribute to these issues as part of a wider response. In addition, AHI has been tasked to ‘*Explore the feasibility of broadening the beef and dairy health programmes and strategies for implementation’* under the banner of animal health programmes [16] as a contribution to a number of wider measures on climate action.

### Methodological limitations

The use of an online platform to elicit opinion provides both advantages in the potential to maximise coverage from diverse stakeholder groups given limited resources, and disadvantages in the collection of relatively superficial information using a survey-type approach which can have limited response rates in certain groups with the potential for bias. Unfortunately, in this study the response rates could not be measured for all respondent types, as participants were anonymous and therefore the nature of any bias remains unknown. In order to overcome these limitations, it is preferable that this work be followed by a form of in-depth interviews or workshops, with key representatives and a focus on future priorities.

Eliciting priorities on one substantive policy domain from different types of stakeholder requires a careful use of surveys as a data-gathering instrument. The work here was informed by the methodological considerations that have been disseminated across the social science community in the form of cognitive aspects of survey methodology (CASM) scholarship (see Tourangeau [41] for a review). CASM informed our survey design in the following three ways. First, we chose our language carefully in our response format, including a *don’t know/not relevant* option, but explicitly omitting a neutral response on the basis that we wanted to elicit more truly held responses rather than more non-committal ones. Although some participant feedback indicated a level of frustration that they could not include a neutral response in their answers, we reasoned that the benefits to be gained from excluding a neutral option outweighed the felt frustration of some respondents. Secondly, we restricted the cognitive burden of participants by only asking them to give three priorities in the second part of the survey: this increased the likelihood that the participants would have processed their responses more deeply than if we had asked them to prioritise a longer list of items. Third, we tried to take account of a participant’s sense of the importance of any one priority by using a weighted response model that adjusted as a function of the number of priorities identified by any one participant.

Almost one third of professional (non-farmer) participants were associated with AHI in some way as working group members. Therefore, these participants are likely more familiar than other respondents with some of the selected non-regulatory bovine health issues under consideration, and of the work of AHI on these issues over the last ten years. Compared to their non-affiliated colleagues, it is plausible that these stakeholders were more likely to indicate that the situation was ‘much better’. However, this positive skew was limited to an average across health concerns of 7.2% of affiliated participants indicating a ‘much better’ situation, which was offset by a decrease of 5.0% in the ‘somewhat better’ categories and an increase of 0.9% in the ‘worse’ categories, compared to non-affiliated colleagues. For simplicity in presenting results, it was decided to keep these participant categories aggregated. Notable outliers to this average relate to BVD (16.9% increase in ‘much better’; 12.4% decrease in ‘somewhat better’), udder health/milk quality (28.9% increase in 'much better'; 16.9% decrease in ‘somewhat better’) and Johne’s disease (increase 7.7% in ‘worse’) when comparing affiliated to non-affiliated participant responses. This bias was therefore not positive in all cases from those individuals who were likely to be well informed. Additionally, despite the positive skew, professional responses were on the whole more conservative than those of farmers.

One confound affects the study design here, that of *self-selection;* by self-selection, it is meant that certain kinds of groups in the universe of potential respondents are overrepresented in the final sample. It is plausible that farmers within the HerdPlus programme are self-selecting into surveys on bovine health as they have already involved themselves in initiatives and programmes to improve bovine health. By extension, this would mean that the survey owners would need to do more to include farmers who may be inferred to be less interested in bovine heath. Due to budgetary and time constraints, systematic efforts to make sure that potentially underrepresented farmer groups were not made. The reasons for the low response rate were also not able to be investigated. The nature of the survey (online) might have precluded those that are less technologically minded and although it was possible to participate on a phone, many participants may have preferred using a computer, requiring desk time – a limited resource for farmers who are primarily active on-farm. The open online nature of the survey with access via a public link may have encouraged multiple responses but these were limited to 1.6% of the total respondents and were unlikely to have had a great influence. For these reasons, the results from this study need to be interpreted with care.

## Conclusion

The aim of this study was to elicit opinion of Irish stakeholders of their perceptions of changes in non-regulatory bovine health issues on their or a typical Irish farm over the last 10 years and of prior issues relevant to non-regulatory bovine health that need to be addressed over the next 10 years. The results are encouraging, demonstrating a perception of improvement in a number of bovine health issues over the last ten years. With respect to the future, the prioritised issues of stakeholders align closely with broader societal concerns, in particular with antimicrobial and anthelmintic resistance, greenhouse gas emissions and animal welfare.

This information is useful to AHI, particularly with respect to prioritised issues for future action. These concerns are broad in scope and will require further considerations, including collaborations, between AHI and partnering organisations. Given that there were differences between farmers and professional services providers in responses, it is useful to consider how the aims and the benefits of future programmes are framed and communicated to all stakeholders. Ideally, one would seek similar views about the aims and benefits of programmes; this makes it easier for programme effectiveness to be advanced. It is also worth considering how the remit of a voluntary collaborative organisation such as AHI is seen to change or indeed is seen to need to change. AHI’s beginnings are clearly traceable to concerns around particularly challenging bovine diseases and conditions, where it was argued that following a best-in-class clinical model would yield positive benefits for animal health and for all stakeholders. It is more contestable for AHI to act in the more ambiguous area of calf/animal welfare, let alone in policies linked to attenuating climate change. Appropriate responses by AHI and other similar organisations to such challenges is in itself a question worth researching.

## Supplementary Information

**Additional file 1.**

**Additional file 2.**

## Data Availability

The datasets used and/or analysed during the current study are available from the corresponding author on reasonable request.

## Abbreviations

AHI: Animal health Ireland
DAFM: Department of Agriculture, Food and the Marine
BVD: Bovine viral diarrhoea
IBR: Infectious bovine rhinotracheitis
SCC: Somatic cell count
UCD: University College Dublin
iNAP: Ireland’s National Action Plan for Antimicrobial Resistance
CAP: Common Agricultural Policy
EU: European Union
ICBF: Irish Cattle Breeding Federation
CASM: Cognitive aspects of survey methodology.
