# Supplementary material for: Stakeholder perceptions of non-regulatory bovine health issues in Ireland: past and future perspectives
Source: Ir Vet J. 2020 Nov 26;73:25. doi: 10.1186/s13620-020-00178-8 (PMC7691078; doi:10.1186/s13620-020-00178-8)
Supplement: Supplementary file 1 — Additional file 1. [file 13620_2020_178_MOESM1_ESM.pdf]

Animal Health Ireland Stakeholder Survey

This survey was presented to individuals online and was dynamic, allowing for skipping irrelevant questions. Farmers were asked to consider questions 'on your farm' for their sector only and professional services providers were asked to consider questions relating to a 'typical farm' for all three sectors.

Animal Health Ireland (AHI) was established in 2009 with the mission to contribute to a profitable and sustainable farming and agri-food sector through improved animal health. The organisation is conducting this survey to explore changes in the perception of a number of health-related conditions over the past ten years and to seek views on health-related challenges for the next decade.

The survey should take no longer than ten minutes to complete. Please answer all questions if possible (all questions marked with an asterix [*] are mandatory). *With some questions (particularly if you are viewing this on a mobile phone), you may need to scroll down to see all possible options.*

Please provide your own personal view when completing the survey; the views of commercial, governmental and representative organisations will be surveyed separately.

1) Background information

1a) How would you best describe yourself?

Choose one only

- Farmer
- Private veterinary practitioner
- State veterinarian
- Agriculture advisory/consultancy/industry
- UCD School of Veterinary Medicine
- Farm relief
- Other

(jumps to description questions, then 6,7,8)

1b) *If farmer…*

Choose one only

- Predominantly dairy *(jumps to 3)*
- Predominantly beef suckler *(jumps to 4)*
- Predominantly beef fattening/finisher *(jumps to 5)*

1c) *If state veterinarian…*

Choose one only

- DAFM
- Local authority
- Other

1d) *If agricultural advisory/consultancy/industry ...*

Choose one only

- Teagasc
- Private

1e) *If UCD School of Veterinary Medicine*

Choose one only

- Staff
- Postgraduate student
- Undergraduate student

2) Contributing to an AHI group

2a) Are you a member of one of Animal Health Ireland's groups: an implementation group (IG) or a technical working group (TWG)?

- Yes
- No

2b) *If yes, please select one of the following*

- IG
- TWG
- IG & TWG

From 1b – addressing dairy farmers

3) **Looking back ... progress on *your farm* over the last 10 years**

The following questions relate to AHI's work to date

3a) Thinking of **BVD** (bovine viral diarrhoea), how have things changed **on your farm** over the last 10 years?

- The health status is much better
- The health status is somewhat better
- The health status is somewhat worse
- The health status is much worse
- Don't know/not relevant

3b) Thinking of **IBR** (infectious bovine rhinotracheitis), how have things changed **on your farm** over the last 10 years?

- The health status is much better
- The health status is somewhat better
- The health status is somewhat worse
- The health status is much worse
- Don't know/not relevant

3c) Thinking of **Johne's disease**, how have things changed **on your farm** over the last 10 years?

- The health status is much better
- The health status is somewhat better

- The health status is somewhat worse
- The health status is much worse
- Don't know/not relevant

3d) Thinking of **udder health & milk quality (the CellCheck programme)**, how have things changed **on your farm** over the last 10 years?

- The health status is much better
- The health status is somewhat better
- The health status is somewhat worse
- The health status is much worse
- Don't know/not relevant

3e) Thinking of **monitoring/feedback of liver and lung lesions at slaughter (the Beef HealthCheck programme)**, how have things changed **on your farm** over the last 10 years?

- The health status is much better
- The health status is somewhat better
- The health status is somewhat worse
- The health status is much worse
- Don't know/not relevant

3f) Thinking of **diseases of young calves**, how have things changed **on your farm** over the last 10 years?

- The health status is much better
- The health status is somewhat better
- The health status is somewhat worse
- The health status is much worse
- Don't know/not relevant

3g) Thinking of **farm biosecurity** (the measures taken to prevent the introduction and spread of infectious diseases), how have things changed **on your farm** over the last 10 years?

- Farm biosecurity is much better
- Farm biosecurity is somewhat better
- Farm biosecurity is somewhat worse
- Farm biosecurity is much worse
- Don't know/not relevant

3h) Thinking of **parasite control**, how have things changed **on your farm** over the last 10 years?

- The health status is much better
- The health status is somewhat better
- The health status is somewhat worse
- The health status is much worse
- Don't know/not relevant

From 1b – addressing beef suckler farmers

4) **Looking back ... progress on *your farm* over the last 10 years**

The following questions relate to AHI's work to date

4a) Thinking of **BVD** (bovine viral diarrhoea), how have things changed **on your farm** over the last 10 years?

- The health status is much better
- The health status is somewhat better
- The health status is somewhat worse
- The health status is much worse
- Don't know/not relevant

4b) Thinking of **IBR** (infectious bovine rhinotracheitis), how have things changed **on your farm** over the last 10 years?

- The health status is much better

- The health status is somewhat better
- The health status is somewhat worse
- The health status is much worse
- Don't know/not relevant

4c) Thinking of **Johne's disease**, how have things changed **on your farm** over the last 10 years?

- The health status is much better
- The health status is somewhat better
- The health status is somewhat worse
- The health status is much worse
- Don't know/not relevant

4d) Thinking of **monitoring/feedback of liver and lung lesions at slaughter (the Beef HealthCheck programme)**, how have things changed **on your farm** over the last 10 years?

- The health status is much better
- The health status is somewhat better
- The health status is somewhat worse
- The health status is much worse
- Don't know/not relevant

4e) Thinking of **diseases of young calves**, how have things changed **on your farm** over the last 10 years?

- The health status is much better
- The health status is somewhat better
- The health status is somewhat worse
- The health status is much worse
- Don't know/not relevant

4f) Thinking of **farm biosecurity** (the measures taken to prevent the introduction and spread of infectious diseases), how have things changed **on your farm** over the last 10 years?

- Farm biosecurity is much better
- Farm biosecurity is somewhat better
- Farm biosecurity is somewhat worse
- Farm biosecurity is much worse
- Don't know/not relevant

4g) Thinking of **parasite control**, how have things changed **on your farm** over the last 10 years?

- The health status is much better
- The health status is somewhat better
- The health status is somewhat worse
- The health status is much worse
- Don't know/not relevant

From 1b – addressing beef fattener/finisher farmers

5) **Looking back ... progress on *your farm* over the last 10 years**

The following questions relate to AHI's work to date

5a) Thinking of **BVD** (bovine viral diarrhoea), how have things changed **on your farm** over the last 10 years?

- The health status is much better
- The health status is somewhat better
- The health status is somewhat worse
- The health status is much worse
- Don't know/not relevant

5b) Thinking of **IBR** (infectious bovine rhinotracheitis), how have things changed **on your farm** over the last 10 years?

- The health status is much better
- The health status is somewhat better
- The health status is somewhat worse
- The health status is much worse
- Don't know/not relevant

5c) Thinking of **monitoring/feedback of liver and lung lesions at slaughter (the Beef HealthCheck programme)**, how have things changed **on your farm** over the last 10 years?

- The health status is much better
- The health status is somewhat better
- The health status is somewhat worse
- The health status is much worse
- Don't know/not relevant

5d) Thinking of **farm biosecurity** (the measures taken to prevent the introduction and spread of infectious diseases), how have things changed **on your farm** over the last 10 years?

- Farm biosecurity is much better
- Farm biosecurity is somewhat better
- Farm biosecurity is somewhat worse
- Farm biosecurity is much worse
- Don't know/not relevant

5e) Thinking of **parasite control**, how have things changed **on your farm** over the last 10 years?

- The health status is much better
- The health status is somewhat better
- The health status is somewhat worse

- The health status is much worse
- Don't know/not relevant

From 1a –addressed to professional service providers

6) **Looking back ... progress on *the typical Irish dairy farm* over the last 10 years**

The following questions relate to AHI's work to date

6a) Thinking of **BVD** (bovine viral diarrhoea), how have things changed **on the typical Irish dairy farm** over the last 10 years?

- The health status is much better
- The health status is somewhat better
- The health status is somewhat worse
- The health status is much worse
- Don't know

6b) Thinking of **IBR** (infectious bovine rhinotracheitis), how have things changed **on the typical Irish dairy farm** over the last 10 years?

- The health status is much better
- The health status is somewhat better
- The health status is somewhat worse
- The health status is much worse
- Don't know

6c) Thinking of **Johne's disease**, how have things changed **on the typical Irish dairy farm** over the last 10 years?

- The health status is much better
- The health status is somewhat better
- The health status is somewhat worse

- The health status is much worse
- Don't know

6d) Thinking of **udder health & milk quality (the CellCheck programme)**, how have things changed **on the typical Irish dairy farm** over the last 10 years?

- The health status is much better
- The health status is somewhat better
- The health status is somewhat worse
- The health status is much worse
- Don't know

6e) Thinking of **monitoring/feedback of liver and lung lesions at slaughter (the Beef HealthCheck programme)**, how have things changed **on the typical Irish dairy farm** over the last 10 years?

- The health status is much better
- The health status is somewhat better
- The health status is somewhat worse
- The health status is much worse
- Don't know

6f) Thinking of **diseases of young calves**, how have things changed **on the typical Irish dairy farm** over the last 10 years?

- The health status is much better
- The health status is somewhat better
- The health status is somewhat worse
- The health status is much worse
- Don't know

6g) Thinking of **farm biosecurity** (the measures taken to prevent the introduction and spread of infectious diseases), how have things changed **on the typical Irish dairy farm** over the last 10 years?

- Farm biosecurity is much better
- Farm biosecurity is somewhat better
- Farm biosecurity is somewhat worse
- Farm biosecurity is much worse
- Don't know

6h) Thinking of **parasite control**, how have things changed **on the typical Irish dairy farm** over the last 10 years?

- The health status is much better
- The health status is somewhat better
- The health status is somewhat worse
- The health status is much worse
- Don't know

7) **Looking back ... progress on *the typical Irish beef suckler farm* over the last 10 years**

The following questions relate to AHI's work to date

7a) Thinking of **BVD** (bovine viral diarrhoea), how have things changed **on the typical Irish beef suckler farm** over the last 10 years?

- The health status is much better
- The health status is somewhat better
- The health status is somewhat worse
- The health status is much worse
- Don't know

7b) Thinking of **IBR** (infectious bovine rhinotracheitis), how have things changed **on the typical Irish beef suckler farm** over the last 10 years?

- The health status is much better
- The health status is somewhat better

- The health status is somewhat worse
- The health status is much worse
- Don't know

7c) Thinking of **Johne's disease**, how have things changed **on the typical Irish beef suckler farm** over the last 10 years?

- The health status is much better
- The health status is somewhat better
- The health status is somewhat worse
- The health status is much worse
- Don't know

7d) Thinking of monitoring/feedback of liver and lung lesions at slaughter **(the Beef HealthCheck programme)**, how have things changed **on the typical Irish beef suckler farm** over the last 10 years?

- The health status is much better
- The health status is somewhat better
- The health status is somewhat worse
- The health status is much worse
- Don't know

7e) Thinking of **diseases of young calves**, how have things changed **on the typical Irish beef suckler farm** over the last 10 years?

- The health status is much better
- The health status is somewhat better
- The health status is somewhat worse
- The health status is much worse
- Don't know

7f) Thinking of **farm biosecurity** (the measures taken to prevent the introduction and spread of infectious diseases), how have things changed **on the typical Irish beef suckler farm** over the last 10 years?

- Farm biosecurity is much better
- Farm biosecurity is somewhat better
- Farm biosecurity is somewhat worse
- Farm biosecurity is much worse
- Don't know

7g) Thinking of **parasite control**, how have things changed **on the typical Irish beef suckler farm** over the last 10 years?

- The health status is much better
- The health status is somewhat better
- The health status is somewhat worse
- The health status is much worse
- Don't know

8) **Looking back ... progress on *the typical Irish beef fattener/finisher farm* over the last 10 years**

The following questions relate to AHI's work to date

8a) Thinking of **BVD** (bovine viral diarrhoea), how have things changed **on the typical Irish beef fattener/finisher farm** over the last 10 years?

- The health status is much better
- The health status is somewhat better
- The health status is somewhat worse
- The health status is much worse
- Don't know

8b) Thinking of **IBR** (infectious bovine rhinotracheitis), how have things changed **on the typical Irish beef fattener/finisher farm** over the last 10 years?

- The health status is much better
- The health status is somewhat better
- The health status is somewhat worse
- The health status is much worse
- Don't know

8c) Thinking of monitoring/feedback of liver and lung lesions at slaughter **(the Beef HealthCheck programme)**, how have things changed **on the typical Irish beef fattener/finisher farm** over the last 10 years?

- The health status is much better
- The health status is somewhat better
- The health status is somewhat worse
- The health status is much worse
- Don't know

8d) Thinking of **farm biosecurity** (the measures taken to prevent the introduction and spread of infectious diseases), how have things changed **on the typical Irish beef fattener/finisher farm** over the last 10 years?

- Farm biosecurity is much better
- Farm biosecurity is somewhat better
- Farm biosecurity is somewhat worse
- Farm biosecurity is much worse
- Don't know

8e) Thinking of **parasite control**, how have things changed **on the typical Irish beef fattener/finisher farm** over the last 10 years?

- The health status is much better
- The health status is somewhat better

- The health status is somewhat worse
- The health status is much worse
- Don't know

From 3,4 – addressed to farmers in dairy and suckler

9 &10) **Looking forward ... challenges facing *your farm* over the next 10 years**

When answering the following questions, please consider the mission of Animal Health Ireland, which is *'to contribute to a profitable and sustainable farming and agri-food sector through improved animal health'*

9a) Please select up to three issues **relevant to your farm** that you think should be priorities for AHI over the next 10 years, to 2030?

Choose up to 3 issues

- Anthelminthic resistance (measures to reduce resistance to fluke and worm treatments)
- Antibiotic resistance (measures to reduce on-farm antibiotic usage and antibiotic resistance)
- Calf welfare
- Clostridial diseases
- Greenhouse emissions (measures to reduce emissions through health-driven efficiencies)
- Infertility
- Lameness
- Mycoplasma bovis

9b) Are there other issues **relevant both to your farm and to AHI's mission** that you feel should be among the top three priorities, please include them here

9c) If yes, list these issues here (maximum 3 please)

From 5 – addressed to farmers in beef fattener/ finisher

11) Looking forward ... challenges facing *your farm* over the next 10 years

When answering the following questions, please consider the mission of Animal Health Ireland, which is *'to contribute to a profitable and sustainable farming and agri-food sector through improved animal health'*

11a) Please select up to three issues **relevant to your farm** that you think should be priorities for AHI over the next 10 years, to 2030?

Choose up to 3 issues

- Anthelminthic resistance (measures to reduce resistance to fluke and worm treatments)
- Antibiotic resistance (measures to reduce on-farm antibiotic usage and antibiotic resistance)
- Clostridial diseases
- Greenhouse emissions (measures to reduce emissions through health-driven efficiencies)
- Lameness
- Mycoplasma bovis

11b) Are there other issues **relevant both to your farm and to AHI's mission** that you feel should be among the top three priorities, please include them here

11c) If yes, list these issues here (maximum 3 please)

From 6,7,8 – addressing professional service providers

12) Looking forward ... challenges facing *Irish dairy farms* over the next 10 years

When answering the following questions, please consider the mission of Animal Health Ireland, which is *'to contribute to a profitable and sustainable farming and agri-food sector through improved animal health'*

12a) Please select up to three issues **relevant to the typical Irish dairy farm** that you think should be priorities for AHI over the next 10 years?

Choose up to 3 issues

- Anthelminthic resistance (measures to reduce resistance to fluke and worm treatments)
- Antibiotic resistance (measures to reduce on-farm antibiotic usage and antibiotic resistance)
- Calf welfare
- Clostridial diseases
- Greenhouse emissions (measures to reduce emissions through health-driven efficiencies)
- Infertility
- Lameness
- Mycoplasma bovis

12b) Are there other issues **relevant both to the typical Irish dairy farm and to AHI's mission** that you feel should be among the top three priorities?

12c) If yes, list these issues here (maximum 3 please)

13) Looking forward ... challenges facing *Irish beef suckler farms* over the next 10 years

When answering the following questions, please consider the mission of Animal Health Ireland, which is *'to contribute to a profitable and sustainable farming and agri-food sector through improved animal health'*

13a) Please select up to three issues **relevant to the typical Irish beef suckler farm** that you think should be priorities for AHI over the next 10 years?

Choose up to 3 issues

- Anthelminthic resistance (measures to reduce resistance to fluke and worm treatments)
- Antibiotic resistance (measures to reduce on-farm antibiotic usage and antibiotic resistance)
- Calf welfare
- Clostridial diseases
- Greenhouse emissions (measures to reduce emissions through health-driven efficiencies)

- Infertility
- Lameness
- Mycoplasma bovis

13b) Are there other issues **relevant both to the typical Irish beef suckler farm and to AHI's mission** that you feel should be among the top three priorities?

13c) If yes, list these issues here (maximum 3 please)

14) Looking forward ... challenges facing *Irish beef fattener/finisher farms* over the next 10 years

When answering the following questions, please consider the mission of Animal Health Ireland, which is *'to contribute to a profitable and sustainable farming and agri-food sector through improved animal health'*

14a) Please select up to three issues **relevant to the typical Irish beef fattener/finisher farm** that you think should be priorities for AHI over the next 10 years?

Choose up to 3 issues

- Anthelminthic resistance (measures to reduce resistance to fluke and worm treatments)
- Antibiotic resistance (measures to reduce on-farm antibiotic usage and antibiotic resistance)
- Clostridial diseases
- Greenhouse emissions (measures to reduce emissions through health-driven efficiencies)
- Lameness
- Mycoplasma bovis

14b) Are there other issues **relevant both to the typical Irish beef fattener/finisher farm and to AHI's mission** that you feel should be among the top three priorities?

14c) If yes, list these issues here (maximum 3 please)

The questionnaire is now completed. Thank you very much for your help. The survey results will be collated and published in due course on the AHI website. For more information on AHI's current programmes and activities, please visit AHI's website.
