# Supplementary material for: Stakeholder perceptions of non-regulatory bovine health issues in Ireland: past and future perspectives
Source: Ir Vet J. 2020 Nov 26;73:25. doi: 10.1186/s13620-020-00178-8 (PMC7691078; doi:10.1186/s13620-020-00178-8)
Supplement: Supplementary file 2 — Additional file 2. [file 13620_2020_178_MOESM2_ESM.pdf]

Additional file 2

Additional summarised results of the survey to elicit stakeholder perceptions of non-regulatory bovine health issues in Ireland.

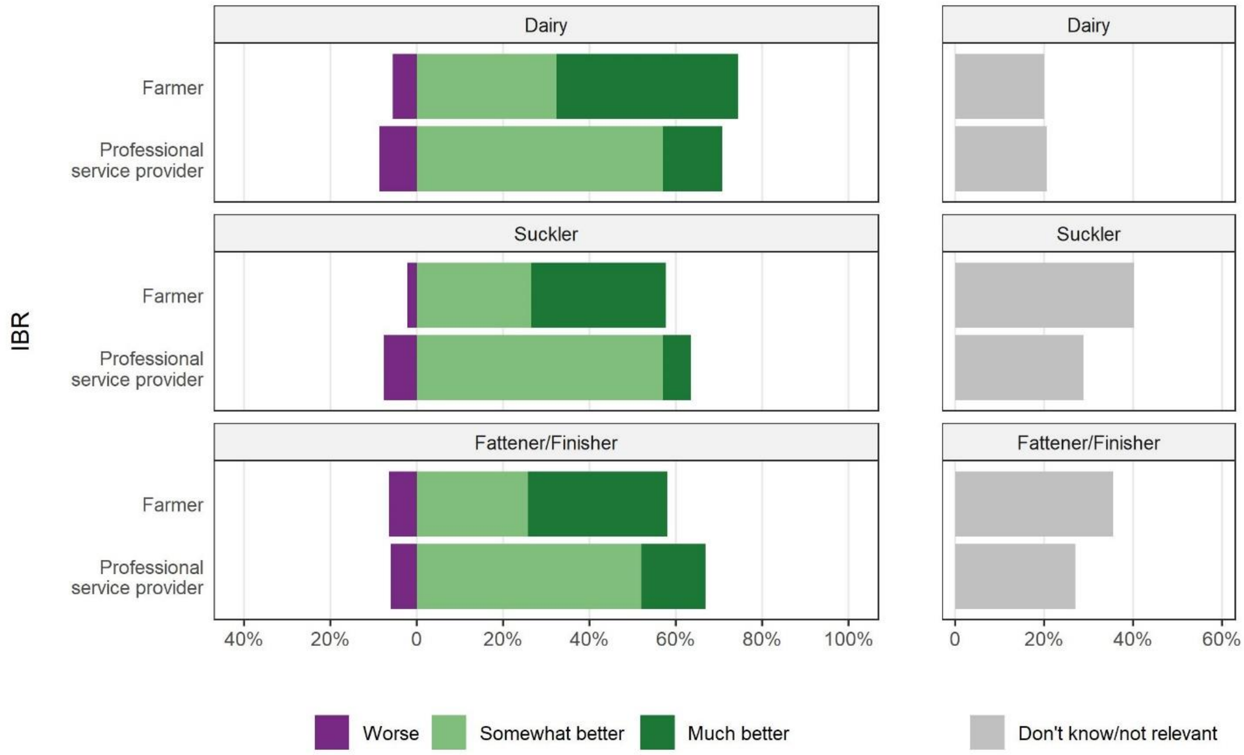

Figure 1: Opinions of Irish farmers and professional service providers of the change over the last 10 years in the status of cattle with respect to IBR, either on their farm (for farmers) or the typical Irish farm (for professional service providers), by sector and type of respondent. For this question, farmers were asked to comment only on the sector they are most associated with, whereas professional service providers were asked to separately comment on all three sectors, dairy, beef suckler and beef fattener/finisher.

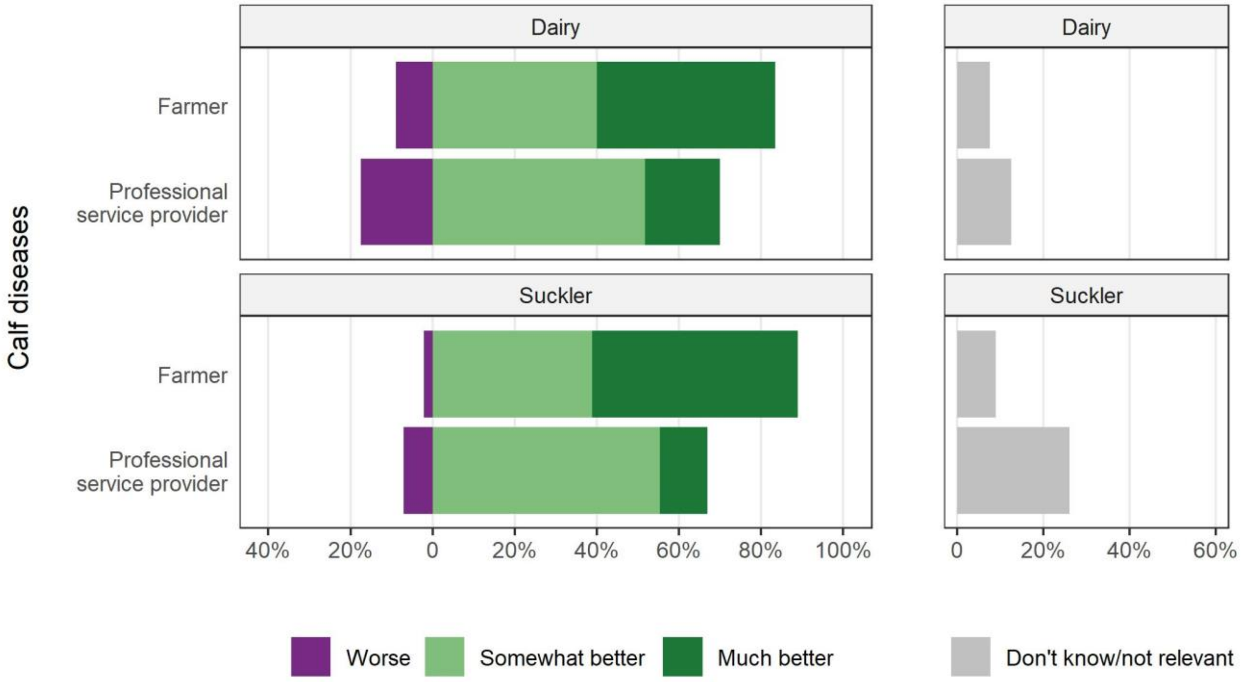

Figure 2: Opinions of Irish farmers and professional service providers of the change over the last 10 years in the status of cattle with respect to diseases of young calves, either on their farm (for farmers) or the typical Irish farm (for professional service providers), by sector and type of respondent. For this question, farmers were asked to comment only on the sector they are most associated with, whereas professional service providers were asked to separately comment on both the dairy and beef suckler sectors.

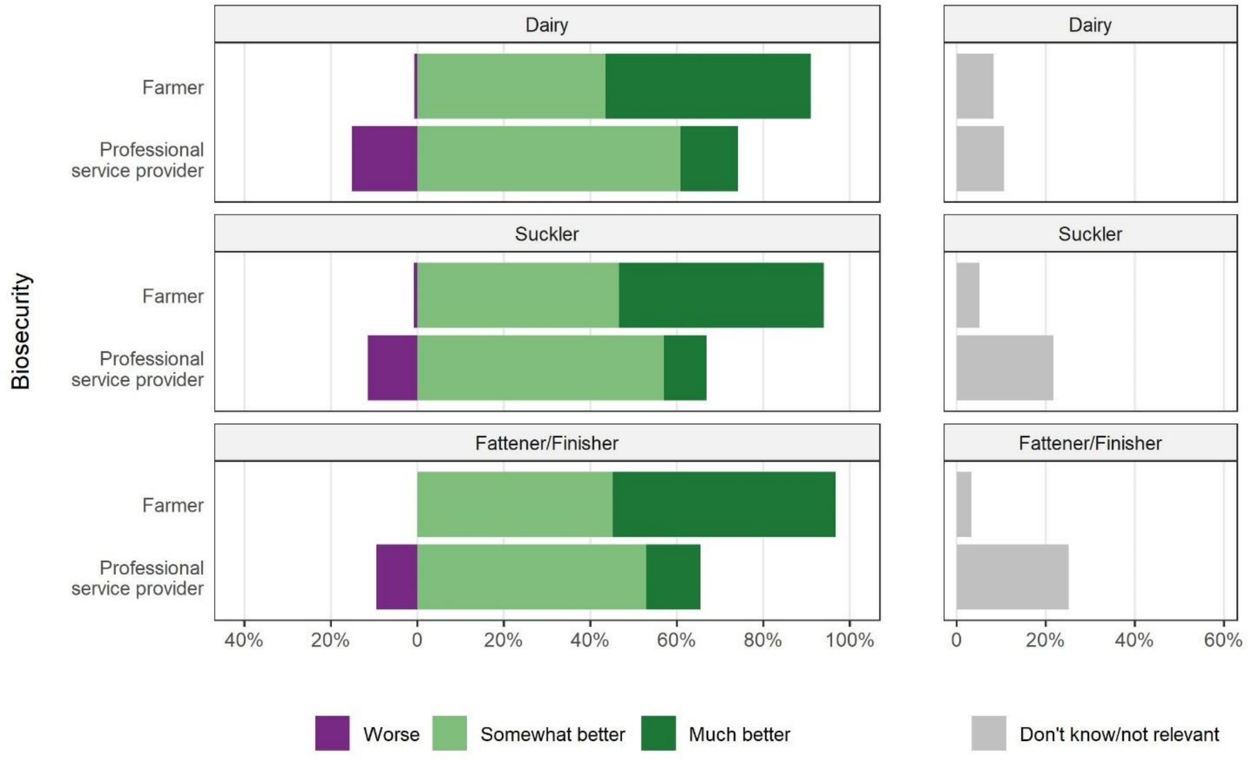

Figure 3: Opinions of Irish farmers and professional service providers of the change over the last 10 years in the status of cattle with respect to farm biosecurity, either on their farm (for farmers) or the typical Irish farm (for professional service providers), by sector and type of respondent. For this question, farmers were asked to comment only on the sector they are most associated with, whereas professional service providers were asked to separately comment on all three sectors, dairy, beef suckler and beef fattener/finisher.

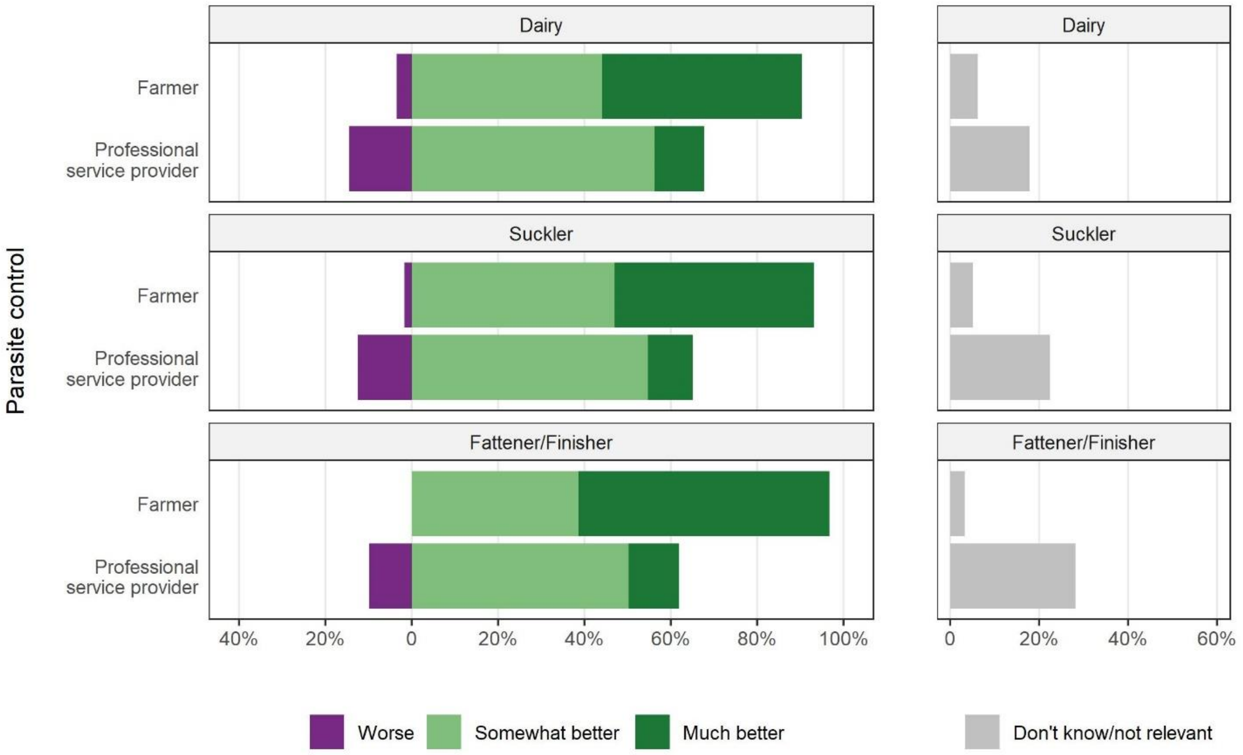

Figure 4: Opinions of Irish farmers and professional service providers of the change over the last 10 years in the status of cattle with respect to parasite control, either on their farm (for farmers) or the typical Irish farm (for professional service providers), by sector and type of respondent. For this question, farmers were asked to comment only on the sector they are most associated with, whereas professional service providers were asked to separately comment on all three sectors, dairy, beef suckler and beef fattener/finisher.
